# Real-World T790M Mutation Frequency and Impact of Rebiopsy in Patients With EGFR-Mutated Advanced Non-Small Cell Lung Cancer

**DOI:** 10.7759/cureus.12128

**Published:** 2020-12-17

**Authors:** Isabel Pereira, Cátia Gaspar, Marta Pina, Isabel Azevedo, Ana Rodrigues

**Affiliations:** 1 Medical Oncology, Instituto Português de Oncologia do Porto Francisco Gentil, EPE, Porto, PRT

**Keywords:** non-small cell lung cancer, t790m, rebiopsy, egfr

## Abstract

Introduction

The T790M resistance mutation is present in about one-half of epidermal growth factor receptor (EGFR)-positive advanced non-small cell lung cancer (NSCLC) patients at disease progression. We aimed to assess the prevalence of this mutation in a real-world setting and the clinical impact of repeated biopsies in its detection.

Methods

This was a single-center retrospective cohort study of patients with EGFR-positive advanced NSCLC diagnosed between 2016 and 2018, who experienced radiographic disease progression during first-line treatment with first- or second-generation EGFR-tyrosine kinase inhibitor (TKI). The frequency of T790M detection and the number of rebiopsies were determined.

Results

A total of 88 patients were included, with a median age of 65 years (range: 38-84 years). The majority of the participants were females (63 (72%)) and non-smokers (70 (81%)). Upon disease progression, 80 (91%) patients were tested for T790M mutation, and the resistance mutation was detected in 57 (71%) cases (58% in plasma samples and 42% in tissue/cytology samples). In 14 (25%) cases, T790M mutation was only detected after rebiopsy (57% by liquid biopsy), which increased the rate of mutation detection in 17%. Subsequent treatment with third-generation EGFR-TKI was possible in 42 (74%) of T790M-positive cases. Detection of T790M mutation was more likely in patients who were less than 65 years old, with EGFR exon 19 deletions and duration of first-line treatment of more than 12 months (p < 0.05).

Conclusions

The frequency of T790M mutation in this study was higher than previously reported, suggesting that repeated biopsies after a negative result are beneficial. This allowed a greater percentage of patients to receive sequential osimertinib in our clinical practice.

## Introduction

According to Global Cancer Statistics, lung cancer remains the leading cause of cancer incidence and mortality in the world, with 2.1 million (11.6%) new cases and 1.8 million (18.4%) deaths in 2018, with more than 80% of the cases attributed to smoking [1]. In Portugal, 5,284 (9.1%) new cases were diagnosed and 4,671 (16.1%) cases died of lung cancer the same year [2].

Around 80 to 85% of lung cancers are non-small cell lung cancers (NSCLCs) [3], and, on average, 33.1% have EGFR mutations, with a higher prevalence in the Asian population [4]. At an advanced stage, these patients have better response rates to epidermal growth factor receptor-tyrosine kinase inhibitor (EGFR-TKI) compared to chemotherapy (ChT) [5]; however, generally, resistance is acquired after 9.2 to 13.6 months of treatment [6-11]. Although there are several resistance mechanisms, the most frequent is the development of T790M mutation, which occurs in approximately 50% of the cases [12,13]. Osimertinib, a third-generation EGFR-TKI irreversible, was initially approved for the sequential treatment of patients who present with T790M mutation and have disease progression with first- or second-generation EGFR-TKI [14-16], and more recently it was approved for the first-line treatment of metastatic NSCLC with EGFR exon 19 deletions or exon 21 L858R mutations, with improved overall survival (OS) [17].

There are limited published data on the Caucasian population with T790M mutation. This study aims to present real-life data on the prevalence of this mutation and the clinical impact of rebiopsy in its detection.

## Materials and methods

A retrospective study was conducted in a Portuguese Oncology Center. Patients with EGFR-positive advanced NSCLC diagnosed between 2016 and 2018 and radiographic disease progression according to the RECIST 1.1 criteria during first-line treatment with first- or second-generation EGFR-TKI were included. Patients who were lost to follow-up were excluded from the study.

The clinical-pathological variables were collected from medical records from treatment initiation with EGFR-TKI until death due to any cause or end of the study (December 30, 2019).

T790M mutation was detected by real-time protein chain reaction using the Cobas® EGFR Mutation Test v2 (Roche Molecular Systems, Pleasanton, CA, USA) in tissue biopsy (fragment or cytology) or liquid biopsy (peripheral blood). When clinical progression was suspected and/or radiographic progression was detected, a rebiopsy by liquid biopsy was performed followed by tissue biopsy if the first sample was negative and if the tumoral lesion was accessible.

The clinical-pathological variables and the number of biopsies were described by frequencies and respective proportions, with the exception of age, and were reported as median and respective intervals. The relationship between the dichotomous categorical variables and the results of the T790M mutation was analyzed using the Fisher’s exact test. Progression-free survival (PFS) 1 and PFS 2 were defined from the beginning of the first- or second-generation EGFR-TKI treatment until radiographic progression of disease or death during the first- or second-line treatments, respectively. OS was defined from the beginning of the first- or second-generation EGFR-TKI until the last observation or death. Data analysis was performed using the Kaplan-Meier method, and the results were compared using the log-rank test. Statistical analysis was performed using SPSS® software version 22 (IBM Corp., Armonk, NY, USA), and the level of significance considered was 0.05.

This study was approved by the Ethics Committee. Due to the retrospective nature of the study, informed consent was waived.

## Results

Of the 159 patients selected by molecular analysis, 88 met the inclusion criteria. The clinical-pathological characteristics of the patients included are described in Table 1. The median age was 65 years (range: 33-84 years), and most of the patients were females (72%) and non-smokers (81%). The most frequent EGFR mutations were exon 19 deletions (65%) and exon 21 L858R (27%). Erlotinib was administrated in 58 (66%) patients, gefitinib in 20 (23%), afatinib in five (6%), and the remaining had to change EGFR-TKI treatment due to unacceptable toxicity. Median PFS 1 was 14 months (95% CI: 11.2-16.7), and the majority (59%) were on treatment for more than 12 months.

**Table 1:** Baseline characteristics of the included patients (n = 88). *Not available in two patients. CNS, central nervous system; EGFR, epidermal growth factor receptor; NOS, not otherwise specified; NSCLC, non-small cell lung cancer; TKI, tyrosine kinase inhibitor

Variables	No. (%)
Gender	
Female	63 (72)
Male	25 (28)
Age (years)	
Median	65
Range	38-84
Performance status*	
0-1	70 (81)
2	11 (13)
3-4	5 (6)
Smoking*	
Non-smoker	70 (81)
Smoker/previous smoker	16 (19)
Histology	
Adenocarcinoma	86 (98)
Adenosquamous	1 (1)
NSCLC NOS	1 (1)
Metastasis	
Pleuropulmonary	58 (66)
Lymph node	40 (46)
Bone	38 (43)
CNS	14 (16)
Liver	13 (15)
Adrenal	9 (10)
EGFR mutation	
19del	57 (65)
L858R	24 (27)
Various	4 (5)
Others	3 (3)
EGFR-TKI	
Erlotinib	58 (66)
Gefitinib	20 (23)
Afatinib	5 (6)
Gefitinib changed to erlotinib	3 (3)
Erlotinib changed to gefitinib	2 (2)

**Figure 1:**
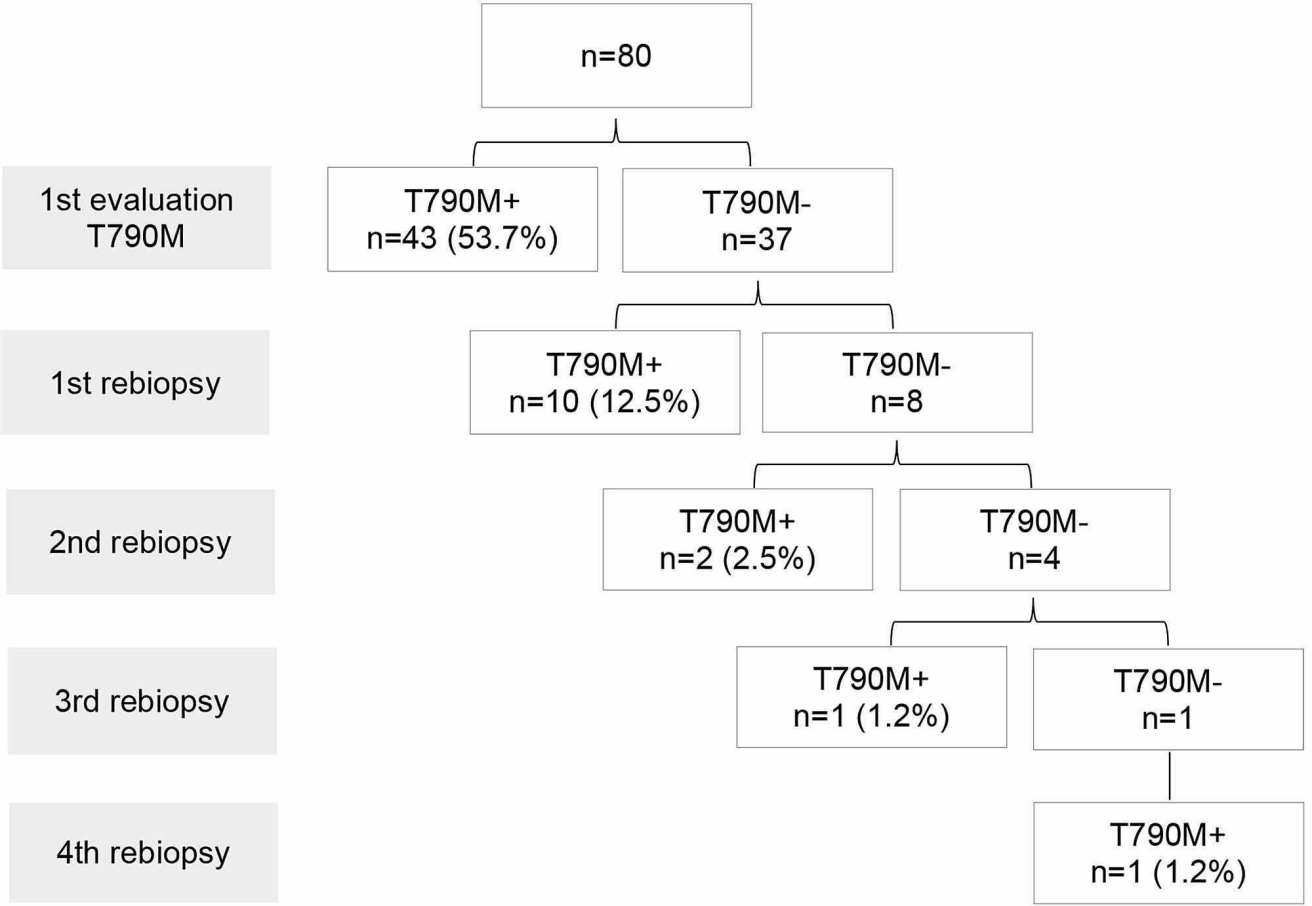
Flowchart of rebiopsies.

During disease progression, eight (9%) patients were not tested for the T790M mutation due to death. The T790M mutation was detected in 57 (71%) cases. A rebiopsy was carried out in 18 patients, enabling the detection of further 14 T790M-positive patients, which means an increment of 17% in the detection rate (Figure 1). In total, 100 liquid biopsies and 42 tissue biopsies were performed, with a positive result in 33 (33%) and 24 (57%) biopsies, respectively. The interval between rebiopsies varied according to the clinician’s decision, and the median time to detection of T790M in the rebiopsy was 21 weeks (range: 0-90 weeks). Median time from imaging progression to the beginning of the second-line treatment was 12 weeks (range: 1-94 weeks).

**Figure 2:**
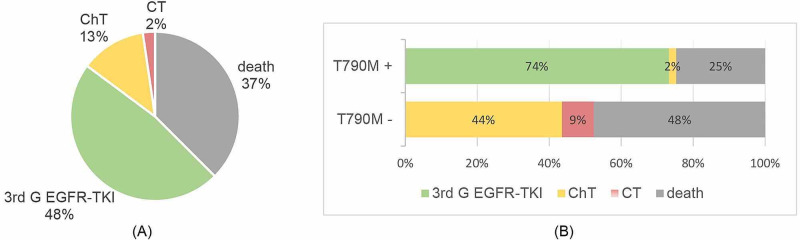
Therapeutic journey of all patients (A) and according to T790M status (B). ChT, chemotherapy; CT, clinical trial; EGFR-TKI, epidermal growth factor receptor-tyrosine kinase inhibitor; G, generation

The therapeutic journey after disease progression is shown in Figure 2. Approximately 37% of the patients did not receive second-line systemic treatment due to clinical deterioration conditioned by rapid disease progression. Of the T790M-positive cases, treatment with osimertinib was possible in 42 (74%) patients, one (2%) patient underwent subsequent treatment with ChT due to difficult access to osimertinib in the current year, and 14 (25%) patients presented rapid disease progression and were therefore proposed for best supportive care. Median time under second-line treatment was four months (95% CI: 1.4-6.5), and median PFS 2 was 26 months (95% CI: 20.1-31.9).

The relationship between clinical-pathological characteristics and T790M status is described in Table 2. T790M mutation was more frequently detected in patients who were less than 65 years of age, with exon 19 deletion and first-line treatment duration of more than 12 months. Other characteristics such as gender, smoking, and the presence of brain metastasis did not show a statistical association with the prevalence of the mutation. In the subgroup with the T790M mutation, there was a tendency toward a higher OS (36 months versus 23 months; p = 0.117), despite not being statistically significant.

**Table 2:** Relationship between clinical-pathological variables and T790M status. *Not available in two patients. **Various and others excluded from the analysis. CNS, central nervous system; EGFR, epidermal growth factor receptor; TKI, tyrosine kinase inhibitor

Variables	T790M		
	Positive No. (%)	Negative No. (%)	p-Value
Gender			
Female	43 (75)	14 (25)	0.275
Male	14 (61)	9 (39)	
Age			
≥65 years	22 (60)	15 (40)	0.047
<65 years	35 (81)	8 (19)	
Performance status*			
0-2	53 (71)	22 (29)	1.000
3-4	2 (67)	1 (33)	
Smoking*			
Non-smoker	47 (73)	17 (27)	0.331
Smoker/previous smoker	8 (57)	6 (43)	
CNS metastasis			
No	50 (73)	19 (27)	0.721
Yes	7 (64)	4 (36)	
EGFR mutation**			
19del	43 (83)	9 (17)	0.019
L858R	12 (55)	10 (45)	
EGFR-TKI			
First generation	53 (71)	22 (29)	1.000
Second generation	4 (80)	1 (20)	
EGFR-TKI treatment time			
≥12 months	42 (82)	9 (18)	0.005
<12 months	15 (52)	14 (48)	

## Discussion

In our sample, the overall frequency of the T790M mutation was 71%, which was higher than that previously reported in other studies [12,13]. This result may be justified by the use of repeated biopsy after a negative result that allowed the mutation to be detected in an additional 17% of the cases. The presence of the T790M mutation would not have been found in 14 of the 57 patients otherwise. A large number of liquid biopsies (100) were performed due to greater ease of access and as it is a less invasive procedure with fewer complications. However, the detection rate of resistance mutation in this type of samples was lower (33%) compared to tissue biopsies (57%). This is consistent with published studies, which revealed that liquid samples appear to have less sensitivity in detecting the T790M mutation, with false-negative rates greater than 30%. This percentage can reach 50% in patients with metastasis limited to the chest [18,19]. Thus, the literature suggests that liquid and tissue biopsies have complementary roles, being recommended during initial plasma research, and, in case of a negative result, are followed by tissue sample analysis [20]. In our clinical practice, patients were able to continue first- or second-generation EGFR-TKI after disease progression without clinical deterioration for a median time of 12 weeks, allowing benefit in improving quality of life compared to ChT.

We consider that the use of rebiopsy had a clinical impact, allowing a greater number of patients, nearly half of our sample, to be eligible for sequential osimertinib. In other studies with real-life data involving non-Asian population, this percentage ranged from 25% to 30% [21,22], similar to that reported in the FLAURA trial in which 85 (31%) of 277 patients allocated to the comparator arm received osimertinib as the first subsequent therapy [17]. However, the percentage of patients who are unable to receive a second-line treatment (37%) due to rapid disease progression is non-negligible and incompatible with the waiting time to schedule a liquid and a sequential tissue biopsy when needed. This proportion is consistent with data from clinical trials with first- and second-generation EGFR-TKI in a first-line setting [23-25]. These data have been taken into account to justify the use of best treatments upfront, such as osimertinib [17].

Studies have shown that prolonged exposure to first- or second-generation EGFR-TKI selects and enriches positive cells for the T790M mutation [26,27], and exon 19 deletion mutations provide a greater tendency toward developing resistance mutation [28]. In our study, the initial EGFR mutation in exon 19 and the EGFR-TKI duration greater than 12 months were also factors associated with a higher probability of having the T790M mutation in our study. On the other hand, the association regarding younger patients (below 65 years of age) is contradictory to the findings reported by most retrospective studies with real-life data [29,30].

Because this is a retrospective data analysis with a small and opportunistic sample, limited to a single center and a short period of time, the results are speculative and should be interpreted with caution.

## Conclusions

In our study, the overall frequency of the T790M mutation in patients who progressed under first- or second-generation EGFR-TKI was approximately 71%. The rebiopsy after a negative result made it possible to increase the number of cases detected with the resistance mutation by 17%. Rebiopsy with tissue sample demonstrated greater sensitivity to detect the mutation compared to peripheral blood.

This study highlights the importance of performing a rebiopsy to detect the T790M mutation in patients with EGFR-mutated NSCLC with disease progression after first- or second-generation EGFR-TKI, allowing a greater number of patients to benefit from sequential treatment with osimertinib.
